# Obesity paradigm and web-based weight loss programs: an updated systematic review and meta-analysis of randomized controlled trials

**DOI:** 10.1186/s41043-021-00240-3

**Published:** 2021-04-08

**Authors:** Leila Jahangiry, Mahdieh Abbasalizad Farhangi

**Affiliations:** 1 Health Education and Health Promotion Department, School of Public Health, Tabriz University of Medical Sciences, Tabriz, Iran; 2 Research Center for Evidence-Based Medicine, Health Management and Safety Promotion Research Institute, Tabriz University of Medical Sciences, Tabriz, Iran

**Keywords:** Internet, Web-based intervention, Obesity, Weight loss

## Abstract

**Backgrounds:**

Web-based therapeutic approaches are new and attractive tools for primary health care systems due to their time and cost-saving nature and their accessibility for different populations. The aim of the current systematic review and meta-analysis is to summarize the results of studies evaluating the effect of web-based interventional programs on weight loss among overweight and obese individuals.

**Methods:**

A literature review from 2000 to 2016 was conducted. Studies were included in the study if they had adult participants with body mass index (BMI) ≥ 25 kg/m^2^, a web-user intervention arm, and a non-web user control arm, with the primary aim of weight loss. Weight change in the interventional group versus control group was pooled with the random-effect model. Data were extracted on sample characteristics, drop-outs, weight loss, intervention duration, and the amount of weight loss. The mean weighted difference and 95% confidence intervals (CI) were calculated.

**Results:**

Eight studies met the inclusion criteria and included in the final model. Overall, using the web-based interventions had a weak non-significant effect on weight loss in overweight and obese individuals (WMD 0.56 kg, CI − 3.474, 4.592; *P* = 0.786). The most important reason was the unadjusted baseline weight of experimental and control groups in included studies, although the stratified analysis showed that, low study quality score and not using feedback and goal-setting in the study were the main factors diminishing the effectiveness of web-based intervention treatment group.

**Conclusion:**

The results of the current meta-analysis indicated no effectiveness of web-based interventional programs in the weight loss of overweight and obese individuals. Although the great between-study heterogeneity and a small number of included studies further highlight the need for additional researches in this field.

## Background

Obesity is a global major health problem and is associated with numerous health issues including cardiovascular disease, several types of cancers, type 2 diabetes, stroke, and musculoskeletal and psychological disorders [1]. According to the 2016 report of the World Health Organization (WHO), the worldwide prevalence of obesity has been more than doubled since 1980 and in 2014, more than 1.9 billion adults were overweight and 600 million adults were obese worldwide [2]. The obesity-associated health complications negatively affect the quality of life and involve high treatment costs for obese individuals while 5–10% weight reduction is associated with reduced risk of health problems and increased health benefits. Strategies to combat obesity and induce weight loss may be pharmacologic, surgical, or behavioral. Among them, behavioral weight-loss strategies are inexpensive, no side effects, and more long-term benefits [3]. Lifestyle interventions for the treatment of overweight and obesity have been effective in achieving moderate weight loss in the short-term. However, it is important to achieve weight loss and to maintain its beneficial effects for the long term. Web-based weight management and weight loss are logical and relatively low-cost approaches to achieve weight loss and maintain it for the long term [4]. Web-based weight-loss interventions have the potential to overcome some of the difficulties of traditional weight loss programs like availability, costs, treatment adherence, and long-term efficacy [5]. Remote health care programs using the internet are considered as a supplemental tool in obesity care through lifestyle modifications [6]. The source information in web-based interventions is accessible 24 h a day and allows individuals to have communication and close contact with a health care professional at any time in a time-saving and cost-effective manner [5]. Considering the above-mentioned beneficial effects of web-based interventional programs to combat obesity, it is important to evaluate the effectiveness of these web-based programs; several review studies are available exploring the effectiveness of web-based weight management programs. However, these studies are limited to studies carried out before April 2011 [6]. The studies included in the final review included both overweight and obese individuals and the aim of the using web was weight loss or weight maintenance. This makes it difficult to have a straight decision about the effectiveness of internet-based behavioral interventions in obesity treatment. Therefore, in the current systematic review and meta-analysis, we aimed to systematically review the results of studies that evaluated the effect of web-based interventional programs on weight loss in obese individuals.

## Methods

This systematic review and meta-analysis focused on the studies aiming at weight reduction delivered to the adult population via web-based programs. The protocol of the current research has been registered by the research undersecretary of Tabriz University of Medical Sciences (Tabriz University of Medical Sciences; IR.TBZMED.VCR.REC.1398.003).

### Search strategy and study selection

Electronic literature search including PubMed, CINAHL, EbscoHost, PsycINFO, Scopus, Ovid, and Science Direct from 2000 to 15 September 2016 was conducted to identify studies investigating the effects of internet-based lifestyle programs on weight loss using the following keywords and various combinations of them in the titles, abstracts, or keywords of the original studies: [internet, computer, phone, smartphone, web-based, telehealth, social media, e-Health, web, online, email, electronic mail, Internet, social networking and obesity, weight loss, body mass index (BMI), overweight, obesity, weight gain, weight change, weight loss, weight management]. We considered articles published in English. We also examined the references list of these publications to identify any additional studies suitable for our purpose.

### Inclusion/exclusion criteria

The studies were included if (1) they were randomized controlled trials (RCT) with a parallel design; (2) participants were overweight or obese adults (defined as BMI ≥ 25 kg/m^2^); (3) participants were aged 18 years or over; (4) the studies consisted of a web-user experimental group and a non-web user control group; (5) the aim of the using the internet was initial weight loss or weight maintenance; (6) participants were apparently healthy individuals and the studies with participants of any chronic or metabolic disease or receiving any pharmacological treatments were excluded. (7) The web-based trials with random allocation of participants and supervised design were included and any web-based intervention that consolidated with other interventions was excluded. (8) The effects of the interventions should be assessed through absolute and/or the percentage change in body weight.

### Data extraction and quality assessment

All potential papers were reviewed by two independent researchers (MAF, LJ) for titles and abstracts of articles for relevance to the topic, and then the full-text of potentially relevant articles were retrieved. The quality control of the articles was performed independently by two authors (MAF, LJ), and any disagreement solved by discussion. Characteristics of studies were abstracted including the name of the first author, publication year, country of origin, journal name, study design, trial duration, and the number of participants in control and intervention groups. Participants’ characteristics consisted of gender, mean age, mean BMI, the weight of participants before and after the intervention, baseline, and after intervention values of BMI. Studies with two independent strata were considered as two studies [7]. The quality of studies was assessed by Jadad scaling algorithm that assigns scores for reported randomization, blinding, and withdrawals [8].

### Data synthesis and statistical analysis

Mean differences and standard deviation (s.d.) of the amount of weight loss in baseline and end of the study in intervention and control groups were considered. If a confidence interval reported in place of s.d., we converted it to s.d. for analyses. The existence of heterogeneity was tested by Cochran’s *Q* test at *P* < 0.05 level of significance. The *I*^2^ test was also used for calculating percent heterogeneity [9]. A fixed-effect model was used for estimating pooled effect sizes. To investigate the source of heterogeneity, a pre-defined subgroup analysis was performed. We assessed trial duration, baseline weight level, and the age of participants. Using meta-regression, we analyzed the contribution of trial duration, baseline weight level, the age of participants, and mean differences of weight loss. Publication bias was analyzed by funnel plot and Egger’s regression asymmetry test [10]. All of the analyses performed using STATA version 12.0 (Stata Corporation, College Station, TX, USA) and *P* < 0.05 was considered significant.

## Results

### Description of the included studies

**Fig. 1:**
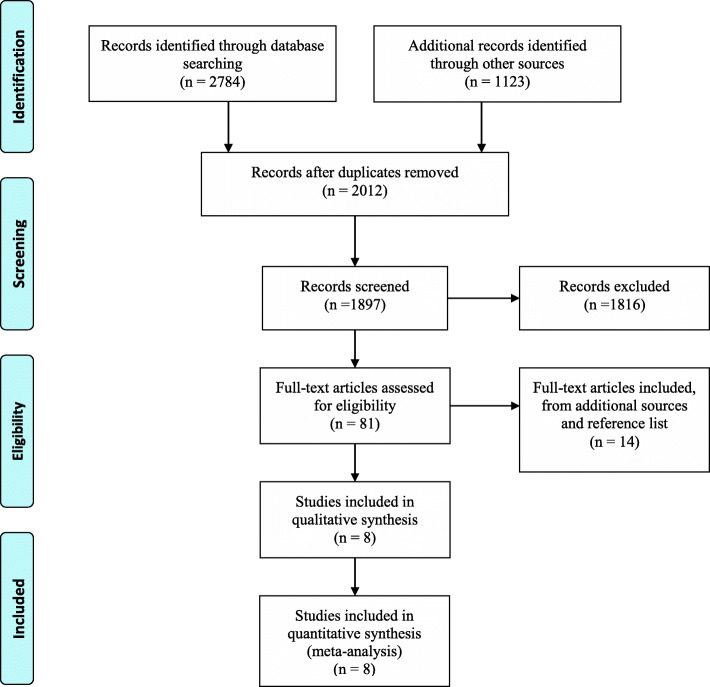
Flowchart of literature search for inclusion in meta-analysis

The flowchart of studies’ selection is presented in Fig. 1. Our electronic search identifies 3907 studies (1903 from PubMed, 544 from Scopus, 1003 from Web of Science, and 457 from science-direct). Of those studies, 1897 citations were retrieved after the exclusion of duplicates. Among them, 1816 studies were excluded based on abstract and title and a total of 81 potentially relevant articles remained for more detailed evaluations. Fourteen additional articles were identified by manual search and were left for full-text review. Of those reviews, 87 articles were excluded and 8 studies met all of the inclusion criteria and were eligible to be included in the final meta-analysis [7, 11–16]. The final eligible trials included a total participant number of 779. Table 1 presents the general characteristics of included studies. The publication year of the articles ranged from 2005 to 2015. From 8 included studies, 7 were included both genders and a single study included only women [16]. The sample size of studies in both intervention and control groups varied between 34 and 203 participants. Duration of intervention in included studies varied between 12 and 24 weeks. All of the included studies were randomized, double-blinded parallel studies, while their outcome of interest was weight loss and was carried out among adults with study-level mean (±s.d.) age of 47 (± 4) years. All of the trials had the study-specific minimum BMI of ≥ 25 kg/m^2^. Included studies had the Jadad score of 2 to 4. Table 2 presents several other qualitative features of web-based interventions included in the current meta-analysis. All of the studies used self-monitoring in their web-based design, 7 studies used intent-to-treat analysis, 4 studies used goal-setting and feedback, 2 studies used social support and self-efficacy, and none of them were tailored web-based interventions.

**Table 1:** Study characteristics of web-based interventions for weight loss in obese and overweight adults

Study	Journal	Setting	Gender	Mean age (years)		Sample size		Length of intervention (weeks)	Jaded score
				Intervention	Control	Intervention	Control		
McDoniel SO [11]	Pat Educ Counsel	USA	Both	45.9	44.9	55	56	12	3
Shaunger SL [7]	Int J Behav Nutr PhysAct	USA	Both	47.7	47.2	49	50	24	4
Hutchesson MJ [12]	Eur J Clin Nutr	Australia	Both	42.6	42.2	99	104	12	4
Mehring M [13]	BMC Family Practice	Germany	Both	46.5	50.9	109	77	12	2
Carr LJ [14]	Prev Med	USA	Both	41.4	49.4	37	30	16	2
Pellegrini CA [15]	Obes	USA	Both	44.1	45.1	17	17	24	2
Park MJ [16]	Int J Med Inform	South Korea	Female	55.8	57.6	42	37	12	2

**Table 2:** The general characteristics of web-based intervention trials included in the meta-analysis

Study	Theory	Goal-setting	Feedback	Self-monitoring	Use of intent-to-treat analysis	Interactive	Tailored	Social support	Self-efficacy
McDoniel SO [11]	Theory of Planned Behavior	Y	N	Y	Y	N	N	N	N
Shaunger SL [7]	TTM and Social Cognitive Theory	N	Y	Y	Y	Y	N	N	N
Hutchesson MJ [12]	Social Cognitive Theory	Y	Y	Y	Y	Y	N	Y	Y
Mehring M [13]	Cognitive Behavioral Therapy, Behavioral Change Theory	Y	Y	Y	Y	N	N	N	N
Carr LJ [14]	Social Cognitive Theory	N	N	Y	Y	Y	N	Y	Y
Pellegrini CA [15]	Not mentioned	Y	Y	Y	Y	N	N	N	N
Park MJ [16]	Internet behavioral theory	N	N	Y	N	N	N	N	N

### The results of meta-analysis of the efficacy of web-based interventions against obesity

**Fig. 2:**
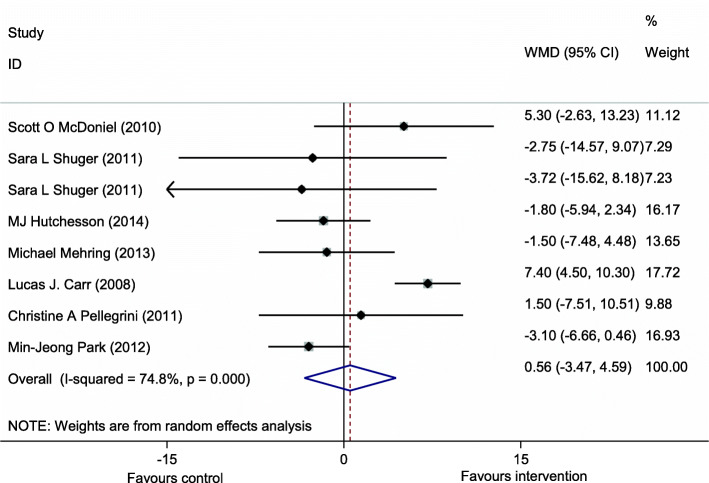
Forest plot illustrating weighted mean difference of the effect of web-based interventional programs on weight loss (test for overall effect: *z* = 0.27 *P* = 0.786; test for heterogeneity *I*^2^ = 74.8, *P* = 0.001)

From eight trials included in the study, only one study showed a significant effect of a web-based intervention on weight loss [14]. The forest plot illustrating the weighted mean difference (WMD) of post-trial weight between intervention and control groups and their 95% confidence interval (CI) is presented in Fig. 2. Test of heterogeneity showed a significant heterogeneity between studies (*I*^2^ = 74.8, *P* = 0.001). Therefore, we used a random effect model to estimate the pooled WMD in the weight loss. Using the random effect model, the pooled effect size of web-based interventions on weight loss was non-significant (0.56 kg, 95% CI − 3.47, 4.59; test for overall effect *z* = 0.27 *p* = 0.786).

### The results of sensitivity analysis

Table 3 shows the results of stratified analysis across the number of several key elements to explore the origin of between-study heterogeneities. As a whole, the effect of a web-based intervention on weight loss is influenced by several characteristics of studies including duration of the study, study quality indices, and participants’ characteristics. According to the results, having an age lower than 45 years old had a negative effect on web-based intervention for weight loss (effect size of 4.19 kg (CI 1.89 to 6.49), *P* < 0.001), whereas having an age of more than 45 had no significant effect on the effectiveness of web-based interventions (effect size of − 0.06 kg (CI 4.21 to 4.08), *P* < 0.97). Surprisingly, using intent-to-treat analysis and interactive web-based programs negatively affected the effectiveness of web-based programs in weight loss (effect size of 3.19 kg (CI 1.18 to 5.21), *P* = 0.002; and 3.81 kg (CI 1.53 to 6.1), *P* = 0.001, respectively). Not including feedback in web-based interventions reduced the effect of a web-based intervention on weight loss and led to an increase in weight (effect size of 3.37 kg (CI 1.21 to 5.54), *P* = 0.002), while using feedback did not affect weight loss. Similarly, not including goal-setting in the web-based programs increased weight (effect size of 2.79 kg (CI 0.62 to 4.96), *P* = 0.012) and having Jadad score of less than 2 increased weight (effect size 2.58 kg (CI 0.53 to 4.63), *P* = 0.014). Moreover, the location of the study was also a significant source of heterogeneity; studies carried out in the USA were associated with an increase in weight (effect size of 5.80 (CI 3.32 to 8.29), *P* = 0.001). While, for studies carried out in other regions, the web-based intervention led to a significant weight loss (effect size of − 2.37 (CI − 4.83 to 0.09), *P* = 0.049).

**Table 3:** Stratified analysis of the effects of web-based interventions on weight loss

Variable	Num. of data	Mean difference (95% CI), kg	***I***^**2**^	***P***_**heterogeneity**_	Significance	Meta-regression
**Mean age**						
< 45	3	4.19 (1.89–6.49)	84.7	0.001	0.000	Referent
≥ 45	4	− 0.06 (− 4.21–4.08)	0.0	0.46	0.97	0.55
Not described	1	− 3.11 (− 6.6–0.46)	–	–	0.088	
**Duration**						
24 months	2	− 0.40 (− 7.58–6.78)	0	0.49	0.91	Referent
16 months	2	6.82 (4.01–9.63)	62.6	0.102	0.000	0.50
12 months	4	− 1.69 (− 4.04–0.65)	16.5	0.31	0.15	0.46
**Use of intent-to-treat analysis**						
No	1	− 3.1 (− 6.6–0.46)	0	–	0.088	Referent
Yes	7	3.19 (1.18–5.21)	68	0.005	0.002	0.005
**Interactive web-based intervention**						
No	4	− 1.35 (− 4.08–1.36)	25.3	0.26	0.33	Referent
Yes	4	3.81 (1.53–6.1)	80.9	0.001	0.001	0.27
**Drop-out rate**						
< 20%	7	1.97 (0.14–3.81)	77.5	0.001	0.03	Referent
≥ 20%	1	− 1.5 (− 7.48–4.81)	0	–	0.62	0.77
**Included feedback in web-based intervention content**						
No	3	3.37 (1.21–5.54)	90.2	0.000	0.002	Referent
Yes	5	− 1.54 (− 4.52–1.43)	0	0.96	0.31	0.83
**Included goal-setting in web-based intervention content**						
No	4	2.79 (0.62–4.96)	86.5	0.001	0.012	Referent
Yes	4	− 0.38 (− 3.3–2.57)	0.0	0.43	0.79	0.56
**Jadad score**						
< 2	4	2.58(0.53–4.63)	86.5	0.001	0.014	Referent
≥ 2	4	− 0.75 (− 4.11–2.61)	0	0.41	0.66	0.27
**Country**						
Others	3	− 2.37 (− 4.83–0.09)	0	0.85	0.049	Referent
USA	5	5.80 (3.32–8.29)	38.80	0.16	0.001	0.004

### The results of meta-regression analysis

We used univariate meta-regression analysis to examine the variation in weight loss effects of web-based interventions attributed to some pre-specified covariates. The univariate meta-regression analysis suggested that only the use of intent-to-treat analysis and country are significant sources of trial heterogeneity (Table 3).

### Publication bias

**Fig. 3:**
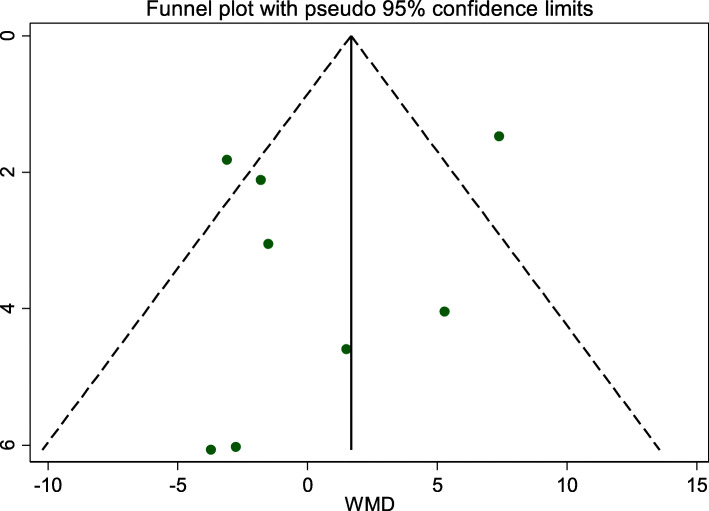
Begg’s funnel plot (with pseudo 95% CIs) of the WMD versus the se (WMD) for studies evaluating the effectiveness of web-based interventions on weight loss

No evidence of publication bias was observed according to Begg’s (*P* = 0.67) and Egger’s (*P* = 0.78) tests did not show any publication bias (Fig. 3).

## Discussion

This meta-analysis systematically reviewed the evidence of the effectiveness of web-based interventions on weight loss. Totally eight studies were included in the current study; these included studies were different from the studies included in the meta-analysis performed by Kodama S [6] that included both overweight or obese participants. We excluded the studies that had outcomes other than weight loss, did not have enough information about web-free control group, initial or final weight of intervention or control group, weight change, or the studies that included overweight individuals. The results of the current meta-analysis indicated that the relative effect of web-based interventional programs on weight loss in obese individuals was non-significant. The possible reason for these findings is the main difference in baseline weight of intervention or control group of studies included. In the current meta-analysis, the forest plot showed weight reduction after web-based interventions in most of the included trials [7, 12, 13, 16]. While in the other three studies, the results were unfavorable and the forest plot indicated inverse findings. In the study by McDoniel S et al. [11], the baseline weights of intervention and control groups were 109 ± 1.9 and 103.8 ± 20.8 while no adjustment for baseline weight was performed. Similarly, no adjustment for baseline weight was also performed in the study by Carr [14] with the baseline weight of 91.1 ± 5 and 83.3 ± 6.6 kg and the study by Pellegrini CA [15] with the baseline weights of 92.3 ± 12.1 and 88.6 ± 12.5 kg for intervention and control groups respectively. Also, the BMI cut-off points of the studies included in the current meta-analysis including both overweight and obese individuals might also be responsible for the non-significant effect of the web-based trials on weight loss in the current meta-analysis. It has been reported that engagement in weight loss programs is in a positive relationship with the level of overweight [17]. However, it seems that overweight and obese individuals are different in the achievement of successful weight loss since behavioral changes and lifestyle modifications are the more preferred method of weight loss among overweight individuals, while obese subjects prefer using anti-obesity medications or even more invasive methods of bariatric surgery [18]. It has been reported that there was a decline in providing lifestyle modification advice and counseling for weight loss to obese individuals over the past 10 years. Reasons behind this reduction, despite increasing obesity levels, are pessimism regarding the potential success of weight loss attempts and increased use of medications to treat obesity-related risk factors and disease, and, perhaps, normalization of excessive body weight [18–20]. Moreover, we excluded the studies in which participants had several other chronic diseases like metabolic syndrome, cardiovascular disease, and hypertension or cancers [21–24], whereas a previous review by Kodama [6] did not exclude such studies. Therefore, this discrepancy between our findings and the finding of the previous review might be attributed to this point. In the current study, not including feedback and goal-setting in the web-based intervention was associated with increased weight or no change in body weight. Moreover, having low study quality reduced the weight loss effect of web-based interventions. Feedback is an effective strategy for encouraging individuals to participate in weight loss and lifestyle modification programs; in this way, subjects are able to monitor the effectiveness of their efforts in weight loss or lifestyle modification [25]. A combination of tailored feedback, tailored information, and goal-setting could increase the effectiveness of web-based educational programs by five folds in weight loss and lifestyle modification [25]. Moreover, multiple feedback strategies are not defined in the reviewed studies, and the effect could not be explained here. Similarly, the studies included in the current review used multiple theories including social cognitive theory, the theory of planned behavior, trans-theoretical model, and cognitive behavioral therapy. Making multiple theoretical frameworks makes us unable to decide whether a particular feedback strategy is more effective compared with others. In most studies, more than one theory was used as a basis for the tailored intervention. Such a problem-driven multiple-theory approach is indeed advocated in recent volumes and articles on applying theory in health education.

## Conclusion

In the current meta-analysis, web-based interventional programs showed a weak non-significant relationship on weight loss. This result was mostly attributed to the unadjusted baseline weight of two included studies, heterogeneity of designs, and low generalization of findings. Moreover, the low-quality score of included trials and not using feedback or goal-setting in the study design were more effective in reducing the effectiveness of web-based trials rather than general characteristics of participants like age and study duration. Future researches should mostly focus on well-designed trials that account for known sources of variation and determine which features of internet-based interventions are critical to achieving success in weight loss.

## Data Availability

The data and the study’s material are available for all if the scientists with reasonable request from corresponding author.

## Abbreviations

BMI: Body mass index
CI: Confidence interval
WHO: World Health Organization
RCT: Randomized controlled trial
SD: Standard deviation
